# Durability of Cutting Tools Obtained by U-FAST Technology in Particleboard Machining

**DOI:** 10.3390/ma18030636

**Published:** 2025-01-31

**Authors:** Joanna Wachowicz, Jacek Wilkowski, Tomasz Dembiczak, Robert Kruzel

**Affiliations:** 1 Institute of Wood Sciences and Furniture, Department of Mechanical Processing of Wood, Warsaw University of Life Sciences, Nowoursynowska Street 166, 02-787 Warsaw, Poland; jacek_wilkowski@sggw.edu.pl; 2 Faculty of Science and Technology, Jan Dlugosz University in Czestochowa, Armii Krajowej Street 13/15, 42-200 Czestochowa, Poland; t.dembiczak@ujd.edu.pl; 3 Faculty of Civil Engineering, Czestochowa University of Technology, Akademicka Street 3, 42-201 Czestochowa, Poland; robert.kruzel@pcz.pl

**Keywords:** cemented carbide, sintering, WC-Co, cutting tools, particleboard machining

## Abstract

The basic material used for tools for machining wood and wood-based materials is WC-Co (Tungsten Carbide with Cobalt)-cemented carbide. The advantages of WC-Co carbides are significant resistance to high temperatures, high hardness, and wear resistance. Wood-based materials, such as particleboard, are particularly difficult to machine due to their considerable inhomogeneity and the presence of various types of hard particle inclusions, such as sand. In addition, unlike metals, wood has a low thermal conductivity, which means that most of the heat generated during milling is transferred to the tool. The consequence of this phenomenon is an increased tool temperature. In addition, the use of a coolant is not possible when machining wood-based materials. The durability of carbide blades is mainly influenced by grain size and cobalt content. When analysing WC-Co as a tool material, it is necessary to consider how it is obtained, as this can also significantly affect its properties. This paper presents the results of a durability study of cutting blades produced by the innovative Upgraded Field-Assisted Sintering Technology (U-FAST) sintering method during particleboard milling. The wear of the blades was measured until the wear value, i.e., the maximum loss at the contact surface VB_max_, was 0.2 mm. Three groups of WC-Co carbides with different WC grain sizes were tested: 0.1, 0.4, and 0.8 µm. Three rotational speeds were used: 12,000, 15,000, and 18,000 rpm. In the machinability tests, blades with a WC grain size of 0.8 µm showed a twofold increase in tool life compared to commercial blades with a similar grain size gradation.

## 1. Introduction

WC-Co carbides are the most widely used tool materials for machining wood-based materials. Their main advantages are their high hardness and relatively high resistance to brittle fracture. Another significant benefit is their low manufacturing cost. These materials are obtained using powder metallurgy technology, which offers a very high degree of freedom in the choice of chemical composition, combining mutually insoluble components [1,2,3,4,5].

The quality of the workpieces, their accuracy, and performance characteristics are significantly impacted by the tools used in machining. The productivity and cost of machining are also affected by the quality of the tool used. The machining of wood is difficult due to the varying density of this material, the presence of defects, and resinification. Machining wood-based materials is strongly influenced by their density and the possibility of sand or other undesirable materials [6,7,8,9].

WC-Co carbides are the most widely used tool materials for manufacturing cutting tools for wood and wood-based materials. They have two main advantages: high hardness and relatively high resistance to brittle fracture. The properties of blades made from WC-Co carbides are influenced by three things: material homogeneity, cobalt content, and WC grain size. Preliminary research definitively shows that the method of obtaining tool materials can influence the performance properties of cutting blades [10,11,12,13,14].

Cemented carbides are sintered using powder metallurgy, a method offering great ease in the choice of chemical composition. It allows the consolidation of mutually insoluble components having extremely different melting points. The technology transforms the compressed powder into a solid material, accompanied by shrinkage, which is macroscopic evidence of changes in the material. In the first stage, the powder to be sintered is subjected to pressing [15,16,17,18]. Then, the moulded part is pushed out of the die and subjected to the sintering process. Conventional sintering involves heating the moulded part using resistance or induction heating furnaces in a reducing or inert atmosphere. The sintering process is carried out in two stages: pre-sintering, followed by finishing and final sintering. The entire process takes several hours. The process is carried out at a high temperature, involving a liquid phase, to obtain a solid material. However, such conditions favour grain growth and thus reduce certain properties (hardness, resistance to brittle fracture) [19,20,21].

FAST (Field-Assisted Sintering Technology) is an alternative to conventional sintering. In these methods, the powder to be sintered is placed in a graphite set: a die and two punches. An electric current flows through the die and punches, constituting a kind of heating element. The heating rate in FAST processes depends on the geometry of the sintering set, its thermal and electrical properties, and the capabilities of the electrical power supply. SPS (Spark Plasma Sintering) is the most widely used FAST technique. In the SPS process, an electrical discharge of a high current but low voltage (in the order of a few volts) is used. The powder to be sintered is placed in a graphite set-up: a die and two punches, which act as a kind of heating element when an electric current flows through it. Its advantages are clear: very short sintering time, homogeneity of the material obtained, and high energy efficiency, mainly due to the direct generation of heat inside the material [22,23,24,25,26,27].

The SPS method undoubtedly has advantages, including the potential to create new materials. Researchers and device designers are still working on upgrading this technology, however. The newly developed U-FAST (Upgraded Field-Assisted Sintering Technology) uses an advanced heating system based on a unique pulse shape, which allows for better results compared to other available FAST devices. The testing of U-FAST materials has shown that it can produce materials that other methods cannot [28,29,30,31].

WC-Co carbides are the most widely used tool materials for machining wood-based materials due to their significant hardness, wear resistance, and durability. Produced via powder metallurgy, these materials offer flexibility in composition and optimize tool performance. Innovations in sintering techniques, such as U-FAST (Upgraded Field-Assisted Sintering Technology), further enhance the properties of WC-Co composites, resulting in longer tool life and more efficient machining. In industrial contexts, WC-Co carbides are not just materials but artefacts—solutions that compress the complexity of machining processes into manageable forms. These artefacts encapsulate accumulated scientific and technical knowledge, allowing users to interact with sophisticated systems without being overwhelmed by the underlying complexities, ultimately improving both productivity and economic efficiency in wood-based material processing [32].

The aim of this study was to carry out durability tests on tools made from WC-Co blanks obtained using the U-FAST method during particleboard machining. Finding the right tool material is important to improve the process. The use of appropriate machining parameters is also an important factor. The aim is to achieve the longest possible tool life. An increase in tool life is significantly associated with an increase in indicators such as productivity or labour efficiency, which in turn is reflected in the competitiveness of manufacturing companies. The scope of the study included the process of forming WC-Co blades and their machinability tests.

## 2. Materials and Methods

### 2.1. Material of Tools

WC-Co carbide sinters were used for the investigation. Samples for testing were obtained from the works [33,34] (Table 1). The purchased powder mixtures were subjected to a sintering process in a U-FAST machine (GeniCore, Warsaw, Poland). The advantage of this method is that the sintering process can be carried out in a very short time. The sintering processes took place over a period of several minutes and were carried out under uniaxial pressure. The sintering temperature was approximately 1220 °C, the holding time was approximately 10 min, and the entire process was carried out under a vacuum of 5 × 10^−2^ mbar. The resulting blanks had a diameter of 56 mm and a height of approximately 2.5 mm.

**Figure 1:**
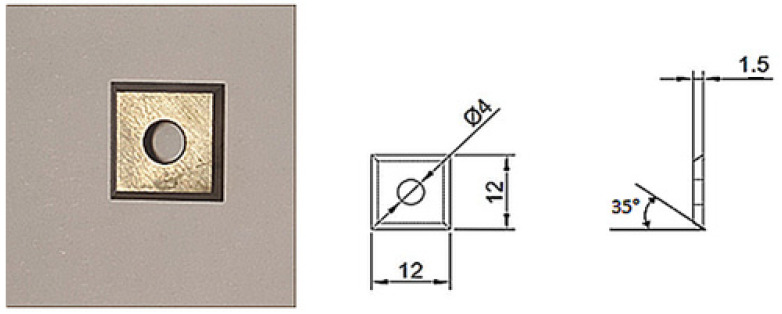
Geometry of the WC-Co cutting tools used for testing.

WC-Co sinters that had been consolidated using the U-Fast method were ground to the required thickness on a surface grinder. Cutting inserts were then obtained using a wire EDM machine (Mitsubishi, Tokyo, Japan) (Figure 1). Following this, the composites were finish-ground and then polished. Significant effort was invested in order to achieve a similar surface roughness to that of the commercial and prepared WC-Co sinters.

### 2.2. Milling Material

**Figure 2:**
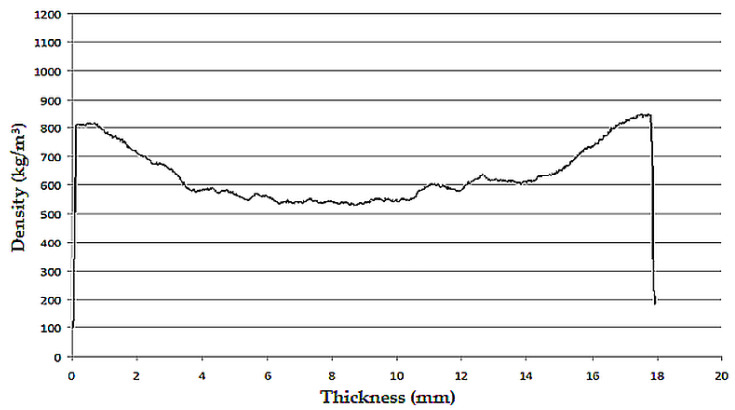
Density profile on a cross-section of a particleboard [11].

The material used for the durability tests was a particleboard panel measuring 1000 × 400 × 18 mm with a density of 648 kg/m^3^. The density profile of the particleboard is shown in Figure 2. Table 2 shows the properties of the particleboard used.

### 2.3. Durability Tests

**Figure 3:**
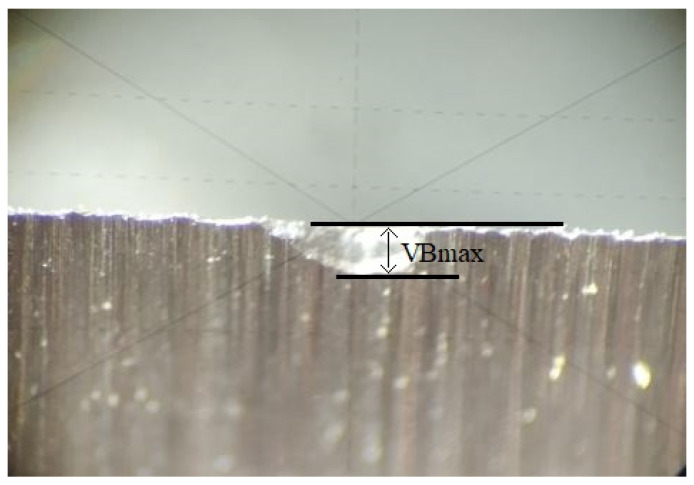
Tool wear. VB_max_ is wear width, which is the tool wear criterion.

Durability tests were carried out on a Busellato Jet 100 machine tool (Thiene, Vicenza, Italy). The knives were mounted in a double-edged arbor cutter. The depth of cut was 4 mm. The machining parameters used are summarised in Table 3. The wear of the contact surface was measured using a workshop microscope every 1 m of feed, corresponding to the length of the plate (Figure 3). The condition of the blade was monitored with the shop microscope. The dulling index was taken to be the dulling bandwidth VB_max_ = 0.2 mm. Prior to the endurance tests, a visual inspection of the leading, trailing, and cutting edge surfaces was carried out to ensure that none of the inserts had any defects in the form of chipping, splitting, or cracking. The wear curves obtained from the tests allowed the tool life values of the inserts to be determined for a blunting index of VB_max_ = 0.2 mm.

## 3. Results and Discussion

**Figure 4:**
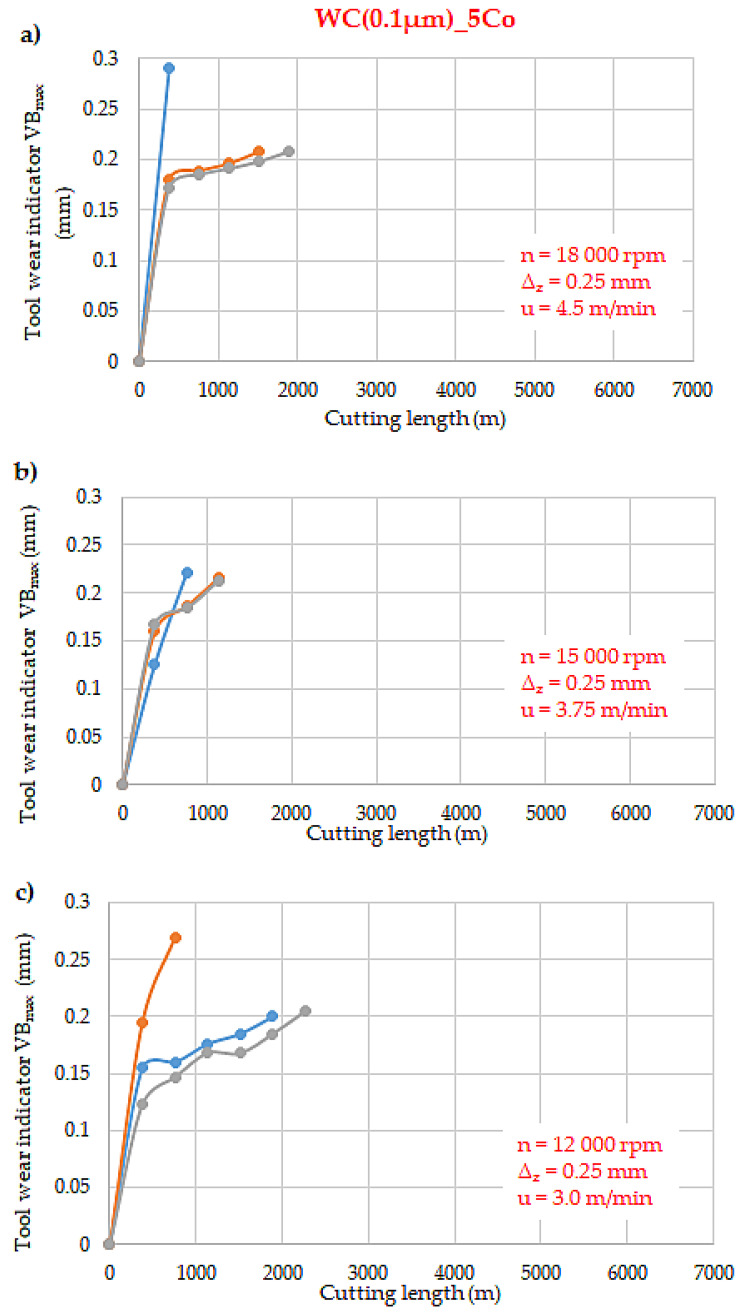
Tool wear curves of WC (0.1 µm)_5Co composites for different cutting speeds: (**a**) *n* = 18,000 rpm, (**b**) 15,000 rpm, (**c**) 12,000 rpm.

**Figure 5:**
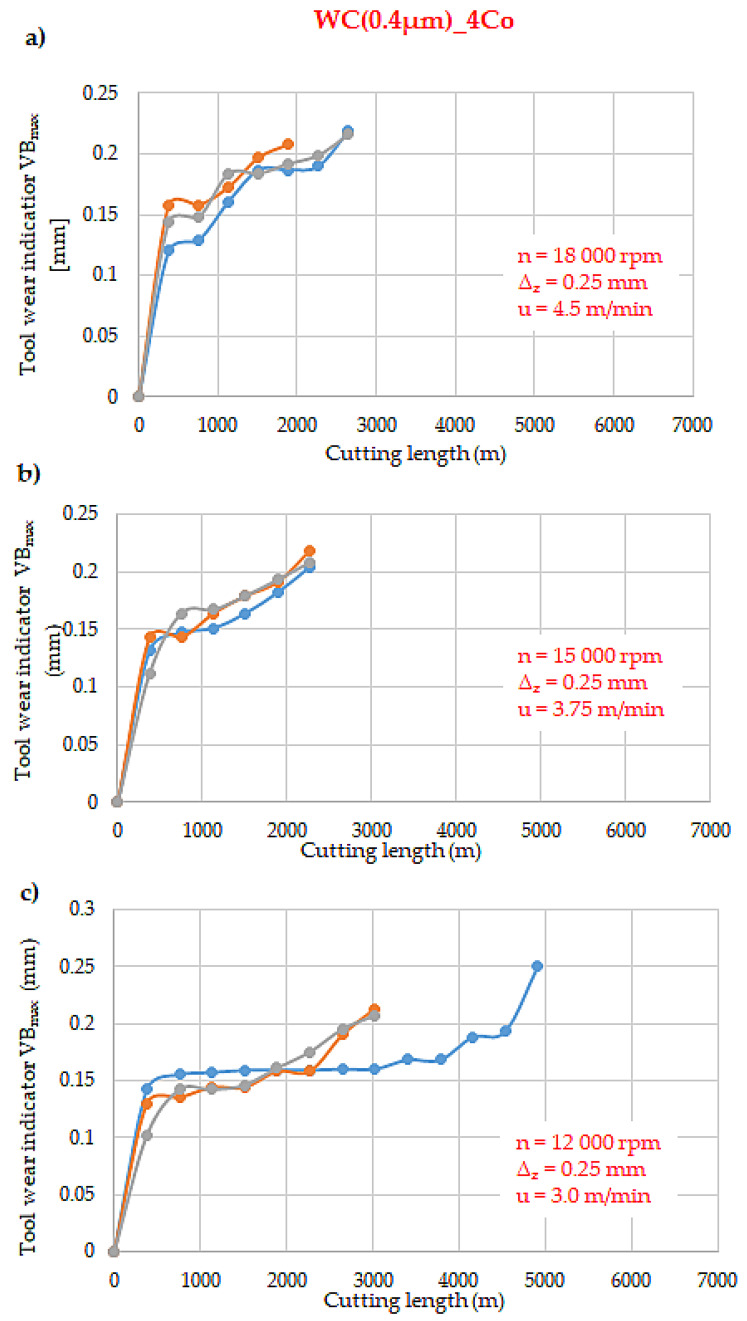
Tool wear curves of WC(0.4 µm)_4Co composites for different cutting speeds: (**a**) *n* = 18,000 rpm, (**b**) 15,000 rpm, (**c**) 12,000 rpm.

**Figure 6:**
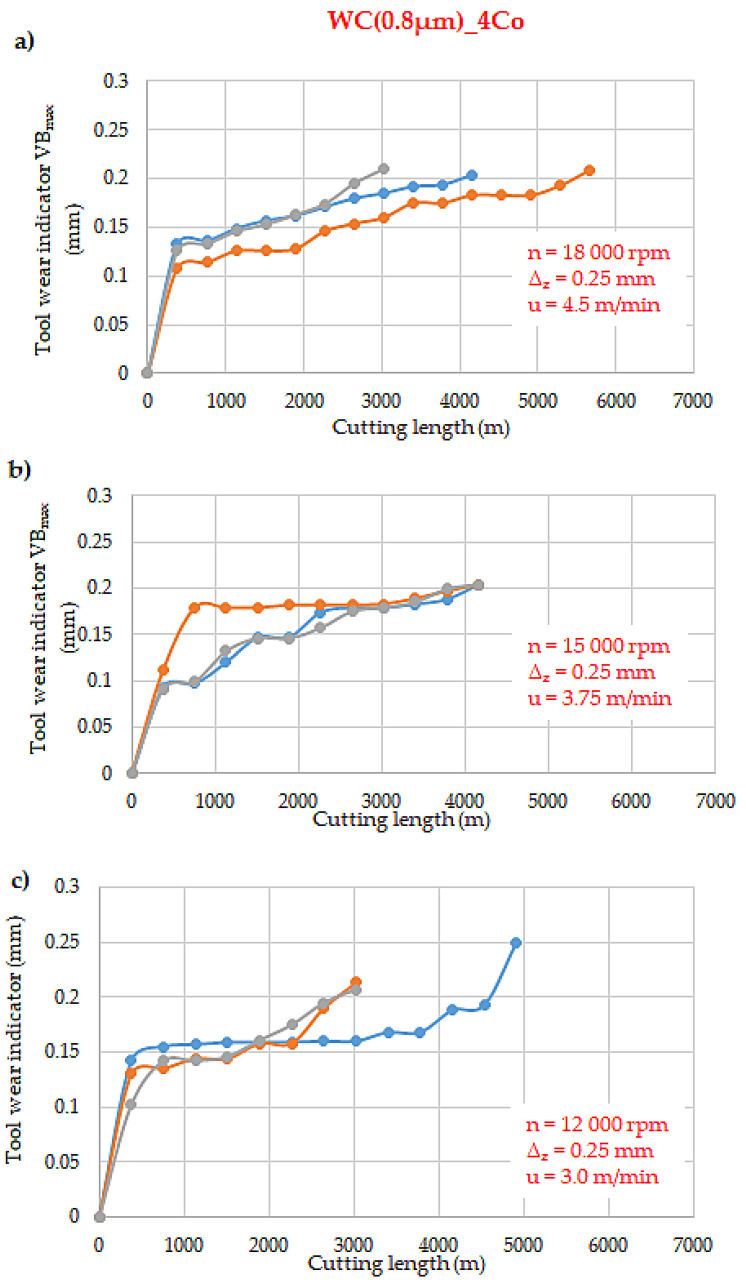
Tool wear curves of WC(0.8 µm)_4Co composites for different cutting speeds: (**a**) *n* = 18,000 rpm, (**b**) 15,000 rpm, (**c**) 12,000 rpm.

Figure 4, Figure 5 and Figure 6 illustrate the wear curves of the WC-Co blades that were tested, obtained using the laboratory U-FAST method. The composites differed in WC grain size, which correlated with different hardness and fracture toughness. In all of the tested blade types, wear followed the Lorenz curve. The wear curves demonstrate three characteristic phases of blade wear: an intensive initial phase (after the first pass of the tool, the wear depth was about 0.1 mm), a stabilised phase, and an accelerated phase.

An analysis of the wear curves revealed that there was an increase in the spread of the wear curves in all WC-Co blade variants that were tested with cutting speeds of 18,000 rpm and 12,000 rpm. The WC(0.1 µm)_(5Co) blades, which possessed the finest WC grain, exhibited the most rapid wear. Concurrently, chipping and rapid cracking were also most prevalent in these blades.

**Figure 7:**
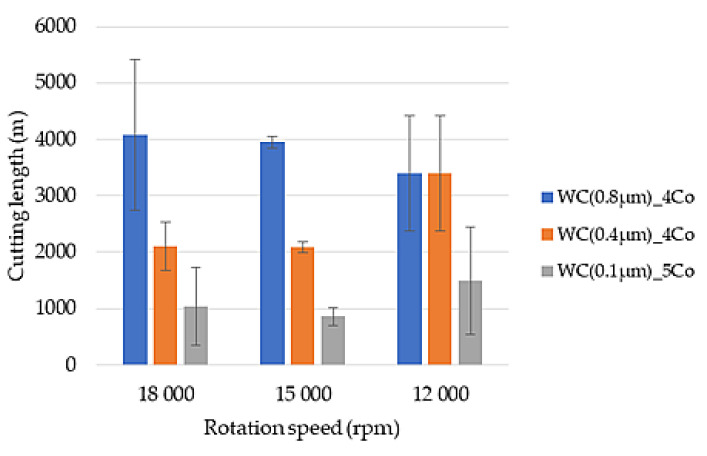
Effect of tool rotation speed on cutting length for Δ_z_ = 0.25 mm, for WC-Co composites.

The wear curves recorded were utilised to ascertain the mean value of the cutting distance for each cutting parameter. Figure 7 presents graphs depicting the mean values of the tool life of blades operating at constant feed per tooth and variable speeds.

As demonstrated in Figure 7, the impact of rotational speed and blade material on blade life, under constant feed per tooth conditions (Δz = 0.25 mm), was investigated. A comprehensive analysis of all three rotational speeds evaluated reveals that WC(0.8 µm)_Co carbides, characterised by a WC grain size of 0.8 µm, demonstrate the greatest longevity. Specifically, when machined at 18,000 rpm, these tools exhibit an average cutting distance of 4079 m. Similarly, at 15,000 rpm, the average distance was 3954 m, which is comparable. However, it is noteworthy that the highest standard deviation was observed at 18,000 rpm, indicating greater variability in the data at this setting.

The enhanced durability exhibited by blades comprising WC grains measuring 0.8 µm is consistent with the observed microstructure in the aforementioned study [33]. The WC grains within these sinters are distinguished by their well-developed edges, while the cobalt is uniformly distributed across their boundaries. Histograms illustrating the grain size distribution in WC(0.8 µm)_Co composites reveal the absence of grain growth during the sintering process. Furthermore, composites with WC grains measuring 0.8 µm exhibited a relative density that exceeded 99% of the theoretical density. Additionally, these composites demonstrated homogeneity, as evidenced by minor deviations from the mean hardness value.

However, for the WC(0.4 µm)_4Co and WC(0.1 µm)_Co blades, the utilisation of the lowest cutting speed of 12,000 rpm resulted in an approximately 50% increase in tool life when compared to the other machining parameters. For cutting speeds of 18,000 and 15,000 rpm, the tool life of the individual blades remained unchanged. The WC(0.4 µm)_4Co blades traversed approximately 2000 m, while the WC(0.1 µm)_Co blades traversed approximately 1000 m.

The WC(0.1 µm)_Co composites were found to demonstrate the lowest durability. In comparison with the blades composed of WC(0.8 µm)_Co composite, the milling distance of the former was reduced by a factor of four at speeds of 18,000 and 15,000 rpm, and by a factor of two and a half at 12,000 rpm.

Furthermore, an analysis of the graph in Figure 7 reveals that at a rotational speed of 15,000 rpm, there is the smallest variation in the obtained durability, and the smallest standard deviation value is recorded.

In paper [35], analogous durability assessments were conducted on commercially procured WC-Co blades, characterised by a WC grain size of 0.5–0.8 µm. Utilising milling parameters of n = 15,000 rpm and Δz = 0.25 mm, the average cutting distance exhibited a similarity to that of WC(0.8 µm)_Co carbides examined in this study, measuring 4061 mm. However, when 12,000 rpm and 18,000 rpm were used, the tool life of the commercial blades was nearly twice as small.

Also as part of the work [35], sintered blades were tested using the typical SPS method. When these were milled at n = 18,000 rpm, an increase in the scatter of the wear curves was observed, as was also found in the present work for all blades obtained using U-FAST technology.

An analysis of the microstructure and material properties of tools obtained by U-FAST technology was conducted in the works [33,34]. The reduced performance properties of WC-Co composites with finer WC grain size were likely influenced by low porosity, which was observed on SEM images of sample fractures, and the presence of a brittle eta phase. This phase was likely responsible for the high hardness and, concomitantly, affected the deterioration of the fracture toughness.

## 4. Conclusions

Blade wear is an inevitable part of material processing. Poor tool performance leads to a loss of productivity and machining quality, and ultimately, a defective tool may fracture, break, lose sharpness, or even fail completely. Tool material and machining parameters are the main factors that affect blade life. Carbide WC-Co is the most commonly selected tool material for machining wood-based materials. On the other hand, an appropriately selected feed rate can ensure optimum working conditions, minimizing wear and improving machining performance.

The research in this study definitively shows the significant influence of the sintering method on the cutting properties of blades made from these. The innovative U-FAST sintering technology is definitely feasible for producing WC-Co-sintered carbides for tools for machining wood-based materials. WC(0.8 µm)_Co blades, milling at a spindle speed of n = 18,000 rpm, had the highest durability. Further research is needed into using different sintering parameters to improve the properties of WC(0.4 µm)_4Co and WC(0.1 µm)_Co composites, and optimizing their chemical composition and operating conditions during chipboard processing.

## Figures and Tables

**Table 1:** Properties of WC-Co composites for particleboard machining tools.

Sample	Grain Size (µm)	Cobalt Content (% wt.)	Hardness (HV30)	K_IC_ (MPam^1/2^)	Literature
WC(0.4 µm)_4Co	0.4	4	2270	8.33	[33]
WC(0.8 µm)_4Co	0.8	4	2085	8.36	[33]
WC(0.1 µm)_5Co	0.1	5	2192	9.27	[34]

**Table 2:** Properties of the particleboard [35].

Wood-Based Board	Density [kg/cm^3^]	Brinell Hardness	Bending Strength [%]	Modulus of Elasticity [MPa]	Sand Content [%]
Three-layer particleboard	649	2.6	8.7	2212	0.185

**Table 3:** Cutting parameters.

Test No.	Spindle Rotation (rpm)	Feed Rate per Tooth, Δ_z_ (mm)	Feed Rate, u (m/min)
P2	18,000	0.25	4.50
P5	15,000	0.25	3.75
P8	12,000	0.25	3.00

## Data Availability

The original contributions presented in this study are included in the article. Further inquiries can be directed to the corresponding author.
